# Molecular detection of *Giardia duodenalis* and the absence of *Cryptosporidium* spp. in owned dogs from Central Vietnam

**DOI:** 10.3389/fpara.2026.1860216

**Published:** 2026-06-02

**Authors:** Vy Thi Tuong Le, Minh-Trang Thi Hoang, Van-Phuong Ngo, Dinh Ng-Nguyen, Tawin Inpankaew

**Affiliations:** 1 Department of Parasitology, Faculty of Veterinary Medicine, Kasetsart University, Bangkok, Thailand; 2 Department of Microbiology and Parasitology, Buon Ma Thuot Medical University, Buon Ma Thuot, Dak Lak, Vietnam; 3 Department of Basic Sciences, Buon Ma Thuot Medical University, Buon Ma Thuot, Dak Lak, Vietnam; 4 Department of Veterinary Medicine, Faculty of Agriculture, Tay Nguyen University, Buon Ma Thuot, Dak Lak, Vietnam

**Keywords:** assemblage D, canine giardiasis, molecular epidemiology, nested PCR, risk factors

## Abstract

**Introduction:**

*Giardia duodenalis* and *Cryptosporidium* spp. are ubiquitous enteric protozoans of significant public health and veterinary concern. Despite their global importance, molecular epidemiological data concerning companion animals in Vietnam remain limited.

**Methods:**

This cross-sectional study evaluated the prevalence, investigated the specific *Giardia* assemblages and *Cryptosporidium* species, and identified risk factors associated with these parasites in 362 owned dogs in Dak Lak province, Central Vietnam. Genomic DNA extracted from fecal samples was screened using nested PCR targeting the small subunit ribosomal RNA (SSU rRNA) locus, followed by sequencing of positive amplicons. Epidemiological risk factors were assessed using multivariable logistic regression.

**Results:**

The overall prevalence of *G. duodenalis* was 8.0% (29/362; 95% confidence interval [CI]: 5.6–11.3%), whereas *Cryptosporidium* spp. DNA was not detected. Sequencing of 18 *Giardia*-positive isolates exclusively identified the canine-specific assemblage D. Multivariable analysis revealed that direct contact with other dogs (adjusted odds ratio [aOR] = 7.55; 95% CI: 1.69–33.82; P = 0.008) and unbathed status (aOR = 0.30; 95% CI: 0.11–0.83; P = 0.021) were significant predictors of infection. Host demographics, location, outdoor access, and deworming history had no significant association with *Giardia *positivity.

**Discussion:**

The exclusive detection of assemblage D indicates that dogs in this rural setting currently pose a minimal direct zoonotic risk to the local human population. These findings emphasize the importance of improving canine management practices, particularly through proper hygiene and the restriction of uncontrolled interactions between dogs, to mitigate transmission risks.

## 1 Introduction

*Giardia duodenalis* and *Cryptosporidium* spp. are ubiquitous foodborne and waterborne protozoan parasites that represent a significant contemporary public health concern (Erickson and Ortega, 2006; Sun et al., 2023). These pathogens inhabit the gastrointestinal tract and are frequently reported causes of diarrhea in humans and various vertebrate hosts (de Lucio et al., 2017; Li et al., 2019; Smith et al., 2020; Yun et al., 2024). Both parasites have a direct life cycle, with transmission occurring via the fecal-oral route, either through direct contact with infected hosts or indirectly via the consumption of food and water contaminated with infective stages (cysts for *G. duodenalis* and oocysts for *Cryptosporidium* spp.) (Agresti et al., 2022; Santin, 2020). These cysts and oocysts are infective immediately upon shedding in feces, are highly persistent in the environment, and require a low infectious dose (Barbosa et al., 2023; Simonato et al., 2017; Sykes, 2023).

*G. duodenalis* and *Cryptosporidium* spp. are among the most common protozoan parasites reported in dogs worldwide (Liao et al., 2020). The worldwide prevalence of *G. duodenalis* in dogs is estimated at 15.6% (Sun et al., 2023), which is notably higher than the 6.0% prevalence of *Cryptosporidium* spp. estimated via molecular diagnostic methods (Taghipour et al., 2020). Symptoms of *Giardia* infection in dogs mainly include acute or chronic intermittent diarrhea; however, the infection is often subclinical, especially in adult animals (Rana, 2024; Sykes, 2023). Canine cryptosporidiosis ranges from asymptomatic to severe diarrhea (Yun et al., 2024), with weight loss and malabsorption more frequently observed in young or immunocompromised dogs (Saari et al., 2019).

Given the rising canine population and the close contact between pets and owners, the zoonotic transmission of these infections is a critical One Health consideration (Adell-Aledón et al., 2018; Barbosa et al., 2023). Globally, *G. duodenalis* and *Cryptosporidium* spp. impose a significant public health burden, with estimated human prevalence rates of 9.7% and 7.6%, respectively (Dong et al., 2020; Sun et al., 2023). Although infections are frequently asymptomatic, they can precipitate severe, life-threatening complications in immunocompromised individuals (Santin, 2020; Szydłowicz et al., 2024).

Both parasites exhibit extensive genetic diversity, resulting in varying zoonotic implications (Li et al., 2025). *G. duodenalis* is classified into eight assemblages (A–H), and humans are primarily infected by the zoonotic assemblages A and B (Adell-Aledón et al., 2018; Gunn and Pitt, 2022). *Cryptosporidium* spp. are generally host-specific, with human cases predominantly attributed to *C. parvum* and *C. hominis* (Barbosa et al., 2023; Gunn and Pitt, 2022). Dogs are frequently documented harboring these zoonotic variants, in addition to their primary, host-adapted *G. duodenalis* assemblages C and D, and *C. canis* (Cai et al., 2021; Taghipour et al., 2020). Notably, these canine-specific genotypes have also been reported in humans, suggesting the risk is not negligible (Barbosa et al., 2023; Li et al., 2025; Yun et al., 2024). Therefore, dogs are considered potential reservoirs (Taghipour et al., 2020), though there is ongoing debate regarding the extent of this zoonotic risk (Smith et al., 2020). While some studies suggest no evidence of direct transmission from dogs to humans (de Lucio et al., 2017; Liao et al., 2020), others support the role of dogs in maintaining zoonotic transmission cycles (Hsu et al., 2023; Julien et al., 2019; Tangtrongsup et al., 2020). This variation may be attributed to differences in target populations and geographic locations.

The tropical climate of Vietnam, marked by high humidity and frequent heavy rainfall, creates optimal conditions for the survival of protozoan cysts and oocysts (Ahmed et al., 2025). Furthermore, agricultural practices in rural areas, such as the use of manure as fertilizer, potentially exacerbate environmental contamination (Iwashita et al., 2021). Previous studies in Vietnam have documented the presence of *G. duodenalis* and *Cryptosporidium* spp. in environmental samples (Nguyen et al., 2016, 2023; Tram et al., 2022), livestock (Cao et al., 2025; Geurden et al., 2008; Nguyen et al., 2012a, 2012b), and humans (Gatei et al., 2003; Iwashita et al., 2021).

There remains a significant scarcity of molecular epidemiological data in Vietnam, particularly regarding companion animals. Existing data on dogs are limited, with previous findings including an 8.6% prevalence of *G. duodenalis* (Nguyen et al., 2018) and the detection of *C. canis* in a single dog (Iwashita et al., 2021). Many previous studies relied on initial microscopic screening, which has lower sensitivity compared to molecular methods and may underestimate the true prevalence. Therefore, this study was conducted to evaluate the prevalence, determine the specific *Giardia* assemblages and *Cryptosporidium* species, and assess risk factors associated with these infections in dogs in Central Vietnam using nested PCR, aiming to elucidate the potential role of dogs in the zoonotic transmission of these parasites.

## 2 Materials and methods

### 2.1 Study area and sample collection

**Figure 1:**
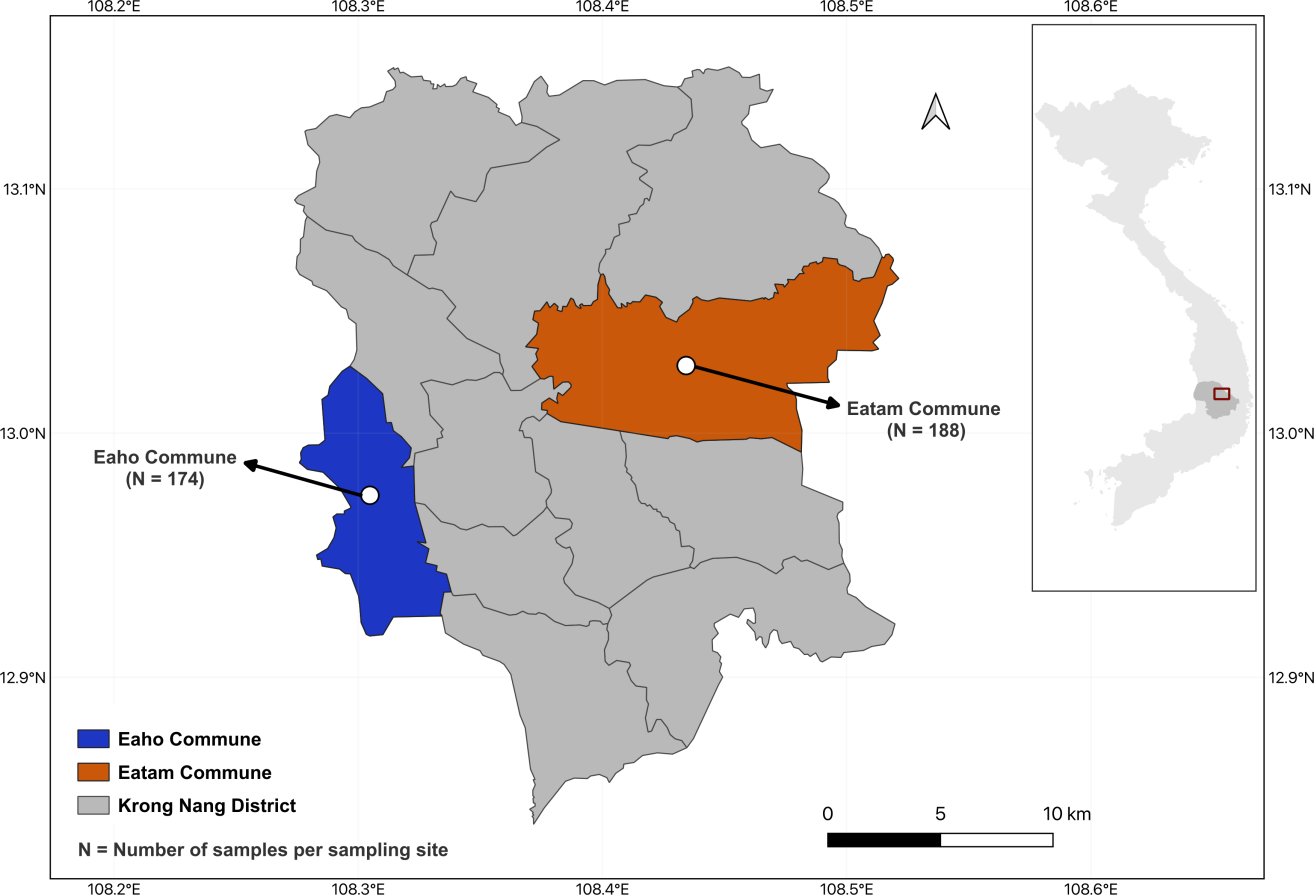
Map of the study area and sampling locations in the Eaho and Eatam communes, Krong Nang district, Dak Lak province, Vietnam. Commune and district borders are shown as they were defined prior to July 2025. Map lines delineate study areas and do not necessarily depict accepted national boundaries.

A cross-sectional study was conducted in Eaho and Eatam communes, Krong Nang district, Dak Lak province, Vietnam, from February to June 2025 (**Figure 1**). Households owning dogs in the selected communes were randomly chosen with the assistance of local authorities, and dogs within these households were then enrolled without restriction by sex, breed, or age.

The minimum sample size was determined using the formula described by Daniel and Cross (2013):


$$n=\frac{Z_{1-\alpha /2\;}^{2}P(1-P)}{d^{2}}$$

The calculation was based on an expected prevalence (*P*) of 8.6% (Nguyen et al., 2018), with a 95% confidence level (*Z* = 1.96) and a margin of error (*d*) of 3%. The final sample size was determined at 362 dogs, adjusting for a 7% anticipated sample exclusion rate.

Approximately 5–10 g of fresh feces was collected from the central portion of the stool of each dog following observed defecation and immediately aliquoted into sterile, labeled 2.0 mL microcentrifuge tubes containing 5% (w/v) potassium dichromate. The samples were transported to the Department of Parasitology, Faculty of Veterinary Medicine, Kasetsart University, for molecular analysis.

Epidemiological data were gathered via interviews with dog owners using a structured questionnaire covering demographic information (age and sex), deworming history, contact with other dogs, outdoor access, and bathing practices.

Ethical approval was granted by the primary academic institution for this research, the Institutional Animal Care and Use Committee of Kasetsart University, in accordance with the ethical guidelines of the National Research Council of Thailand (no. ACKU67-VET-098). The study was conducted with explicit permission from Krong Nang district authorities and informed consent from all dog owners prior to sample and data collection.

### 2.2 DNA extraction

Genomic DNA was extracted from approximately 200 mg of each fecal sample using the E.Z.N.A.^®^ Stool DNA Kit (Omega Bio-Tek, Norcross, GA, USA) according to the manufacturer’s instructions. The DNA was eluted in 100 µL of elution buffer. The concentration and purity of the extracted DNA were measured using a NanoDrop™ One Microvolume UV-Vis Spectrophotometer (Thermo Fisher Scientific, Waltham, MA, USA), and the DNA extracts were stored at -20 °C until further analysis.

### 2.3 Molecular detection and genotyping

Molecular detection of *G. duodenalis* and *Cryptosporidium* spp. was performed on all genomic DNA samples using nested PCR targeting the small subunit ribosomal RNA (SSU rRNA) gene. All primer sequences used in this study are listed in **Table 1**.

**Table 1:** Oligonucleotide primers and cycling conditions used for the molecular detection and genotyping of *G. duodenalis* and *Cryptosporidium* spp. targeting the SSU rRNA gene.

Primer name	Primer sequences (5’-3’)	Cycling conditions	Size (bp)	Reference
***G. duodenalis***		95 °C/15 min (95 °C/15 s 58 °C/30 s 72 °C/1 min) ×35 72 °C/7 min		
RH11	CATCCGGTCGATCCTGCC		292	(Hopkins et al., 1997)
RH4	AGTCGAACCCTGATTCTCCGCCAGG			
GiarF	GACGCTCTCCCCAAGGAC		174	(Read et al., 2002)
GiarR	CTGCGTCACGCTGCTCG			
***Cryptosporidium* spp.**		95 °C/15 min (94 °C/45 s 55 °C/45 s 68 °C/1 min) ×35 68 °C/7 min		
SSU-F1	TTCTAGAGCTAATACATGCG		1325	(Xiao et al., 1999)
SSU-R1	CCCATTTCCTTCGAAACAGGA			(Xiao et al., 2000)
SSU-F2	GGAAGGGTTGTATTTATTAGATAAAG		840	(Xiao et al., 1999)
SSU-R2	CTCATAAGGTGCTGAAGGAGTA			(Jiang et al., 2005)

PCR mixtures were prepared in a final volume of 20 µL, containing 4 µL of HOT FIREPol^®^ Blend Master Mix Ready to Load, 5× (Solis BioDyne, Tartu, Estonia), 1 µL of each primer (10 µM), 12 µL of nuclease-free water, and 2 µL of genomic DNA for the primary PCR, or the primary PCR product for the nested reaction. Genomic DNA from dogs with confirmed *G. duodenalis* and *Cryptosporidium* spp. infections were included in each run as a positive control, and nuclease-free water as a negative control. All reactions were performed on a T100™ Thermal Cycler (Bio-Rad Laboratories, Hercules, CA, USA).

PCR products were visualized by electrophoresis on a 1.5% agarose gel. Positive amplicons were excised and purified using the MEGAquick-spin™ Plus DNA Purification Kit (iNtRON Biotechnology, Seongnam, South Korea). The concentration of purified products was measured using a NanoDrop™ One Microvolume UV-Vis Spectrophotometer (Thermo Fisher Scientific, Waltham, MA, USA).

Purified amplicons were sequenced using the unidirectional Sanger method by U2Bio (Seoul, South Korea). Nucleotide sequences and chromatograms were inspected using SnapGene^®^ Viewer version 8.2.2 (Dotmatics, Boston, MA, USA). Consensus sequences were compared with reference sequences available in the GenBank database using the Basic Local Alignment Search Tool (BLAST; https://blast.ncbi.nlm.nih.gov/Blast.cgi).

### 2.4 Statistical analysis

Data were organized in Microsoft Excel and analyzed using IBM SPSS Statistics version 27 (IBM Corp., Armonk, NY, USA). Prevalence was estimated with 95% confidence intervals (CIs) using the Wilson score method. Pearson’s Chi-square or Fisher’s exact tests were performed for univariable analysis. Variables with *P* < 0.25 were analyzed via backward stepwise multivariable logistic regression. Final results are reported as adjusted odds ratios (aOR) with 95% CIs. Associations were considered statistically significant at *P* < 0.05.

## 3 Results

### 3.1 Molecular detection and genotyping of *G. duodenalis* and *Cryptosporidium* spp.

A total of 362 fecal samples were analyzed in this study. Based on nested PCR amplification of the SSU rRNA gene, the overall prevalence of *G. duodenalis* infection was 8.0% (29/362; 95% CI: 5.6–11.3%). In contrast, *Cryptosporidium* spp. DNA was not detected in any of the examined samples (0/362; 95% CI: 0.0–1.1%).

Amplicons of sufficient quality for sequencing were successfully obtained for 18 of the 29 *Giardia*-positive samples (62.1%). BLAST analysis showed that all 18 sequences shared 100% nucleotide identity with *G. duodenalis* assemblage D (GenBank accession no. JQ245138).

### 3.2 Risk factors associated with *G. duodenalis* infection

The results of the univariable and multivariable logistic regression analyses are summarized in **Table 2**. In the final multivariable model, contact with other dogs and bathing practices were identified as significant predictors of infection. Dogs with direct contact with other dogs had 7.55 times higher odds of *Giardia* positivity compared to those without such contact (aOR = 7.55; 95% CI: 1.69–33.82; *P* = 0.008). Bathing practices were significantly associated with infection status, where unbathed dogs had lower odds of infection than their bathed counterparts (aOR = 0.30; 95% CI: 0.11–0.83; *P* = 0.021). Although outdoor access passed the initial univariable screening, it was not retained in the final model (*P* = 0.833). Location, age, sex, and deworming history were not significantly associated with *Giardia* infection.

**Table 2:** Risk factors associated with *G. duodenalis* infection in owned dogs in Dak Lak province, Vietnam (n = 362).

Variable	No. examined	No. positive	Prevalence	Univariable analysis	*P-*value	Multivariable analysis	*P-*value
	(N)	(n)	(%)	cOR (95% CI)		aOR (95% CI)	
Location							
Eaho	174	16	9.2	Ref.			
Eatam	188	13	6.9	0.73 (0.34–1.57)	0.425		
Age group							
> 12 months	216	15	6.9	Ref.			
≤ 12 months	146	14	9.6	1.42 (0.66–3.04)	0.363		
Sex							
Female	207	14	6.8	Ref.			
Male	155	15	9.7	1.48 (0.69–3.16)	0.312		
Outdoor access							
No	88	3	3.4	Ref.		—	—
Yes	274	26	9.5	2.97 (0.88–10.06)	0.068	—	—
Contact with other dogs							
No	101	2	2.0	Ref.		Ref.	
Yes	261	27	10.3	5.71 (1.33–24.48)	0.009	7.55 (1.69–33.82)	0.008**
Deworming history							
Yes (≤ 12 months ago)	94	6	6.4	Ref.			
No/Never	268	23	8.6	1.38 (0.54–3.49)	0.499		
Bathing practice							
Yes	43	6	14.0	Ref.		Ref.	
No	319	23	7.2	0.48 (0.18–1.25)	0.135^a^	0.30 (0.11–0.83)	0.021*

*P < 0.05, **P < 0.01. ^a^*P*-value calculated using Fisher’s exact test. Dashes (—) indicate that the variable was not retained in the final parsimonious model following backward stepwise elimination (Wald *P* > 0.05). cOR, crude odds ratio; aOR, adjusted odds ratio.

## 4 Discussion

Accurate diagnosis of *G. duodenalis* and *Cryptosporidium* spp. is essential for both veterinary and public health (Abdelaziz and Sorour, 2021; Jian et al., 2024). *G. duodenalis* was detected in 8.0% of the sampled dogs. This rate is highly comparable to previous reports from Vietnam (8.6%) (Nguyen et al., 2018), China (9.4%) (Li et al., 2019), and Egypt (8.5%) (Abdelaziz and Sorour, 2021). While a lower prevalence (3.0%) was reported in Thailand (Khine et al., 2021), exceptionally high infection rates have been documented in European countries, such as Italy (41.1%) (Agresti et al., 2022), Spain (36.5%) (Adell-Aledón et al., 2018), and Portugal (33.8%) (Pereira et al., 2021). In the present study, *Cryptosporidium* spp. DNA was not detected. This finding aligns with the low prevalence of 0.7% observed in stray dogs in Thailand (Khine et al., 2021). Globally, the prevalence of *Cryptosporidium* spp. in companion animals is heterogeneous but typically remains below 10% across North America, Europe, the Western Pacific, and Asia (Nahar et al., 2026; Taghipour et al., 2020). In Asia, infection rates have been documented at 4.2% in Bangladesh (Nahar et al., 2026), 6.9% in China (Li et al., 2019), and 8.4% in Taiwan (Hsu et al., 2023). These prevalence variations for *G. duodenalis* and *Cryptosporidium* spp. can be attributed to environmental conditions, seasonality, host demographics, and the diagnostic methods employed (Liao et al., 2020; Mugala et al., 2018; Yun et al., 2024).

Determining the specific assemblages of *G. duodenalis* is critical for elucidating its zoonotic potential (Pereira et al., 2021). In this study, all successfully sequenced isolates were identified as the canine-specific assemblage D. This finding is consistent with global trends, where assemblages C and D predominate in dog populations (Nahar et al., 2026; Sun et al., 2023), as well as with previous data from Vietnam (Nguyen et al., 2018) and China (Li et al., 2019). While studies in Thailand also report a predominance of canine-specific genotypes, they have concurrently documented the presence of the zoonotic assemblage A (Khine et al., 2021). Since only assemblage D was detected, the direct zoonotic risk to the local human population appears minimal (Nguyen et al., 2018). However, this risk cannot be disregarded, as human infections with canine-specific assemblages C and D have been documented (Sun et al., 2023).

In the present study, direct contact with other dogs was a significant risk factor for *Giardia* infection, while outdoor access was not. In these rural communes, owned dogs with outdoor access are unrestrained and roam freely. This finding regarding rearing environment aligns with Sun et al. (2023) and Nahar et al. (2026), reporting no significant difference in *Giardia* prevalence between free-roaming and indoor dogs. Furthermore, the relatively low *Giardia* prevalence (8.0%) observed in this study suggests that widespread environmental contamination is limited. Thus, transmission in this population might be driven primarily by dog-to-dog interactions rather than general environmental exposure. Since *Giardia* cysts are immediately infective upon excretion (Simonato et al., 2017), direct contact between animals constitutes a primary pathway for fecal-oral transmission (Yu et al., 2017). Bathed dogs demonstrated higher odds of infection than unbathed dogs. Bathing might act both as an epidemiological proxy for prior exposure and as a mechanical driver of transmission. Owners typically wash dogs reactively, often following fecal soiling, indicating heavy prior exposure. While routine bathing is recommended to remove fecal debris and prevent reinfection from cysts on the fur (Saleh et al., 2016), the subsequent drying phase can create a transmission risk. The general practice in these rural settings is to allow bathed dogs to dry naturally. Since *Giardia* cysts can survive longer in moist environments, this prolonged dampness maintains cyst viability on the hair coat. Given the low infectious dose of *Giardia*, requiring as few as 10 cysts to cause infection (Ciuca et al., 2021; Godínez-Galaz et al., 2019), this moisture retention may increase the risk of infection during self-grooming. This improper drying interacts with conspecific social behavior to facilitate transmission. When unrestrained dogs with cyst-contaminated damp coats engage in social interactions, these viable cysts can pose an infection risk for interacting dogs. Transmission may occur through natural conspecific behaviors, including sniffing, licking, mutual grooming, and playful biting. Effective management of *Giardia* infection requires an integrated approach (Saleh et al., 2016). Owners should be advised to wash their dogs using appropriate antiprotozoal shampoos (e.g., containing chlorhexidine digluconate) (Ciuca et al., 2021) and to thoroughly dry the fur to facilitate cyst desiccation. This hygiene protocol, combined with targeted therapeutics (e.g., fenbendazole), and the restriction of animal interactions is recommended to reduce the risk of transmission.

Deworming history showed no statistically significant association with *Giardia* infection, consistent with findings by Nahar et al. (2026). This finding is likely due to the highly variable timing of prior treatments. While Sun et al. (2023) reported that deworming within the previous month significantly reduced infection rates, our study recorded deworming history over a broader span of up to 12 months prior to sampling. Given the frequent interaction between dogs in these rural communes, there are ongoing opportunities for exposure over time. An anthelmintic treatment administered several months prior would no longer offer direct protection. Therefore, deworming history over this extended timeframe was not a significant predictor of infection. Demographic variables (sex and age) were not significantly associated with *Giardia* infection. The lack of a sex association is widely reported (Abdelaziz and Sorour, 2021; Adell-Aledón et al., 2018; Hsu et al., 2023; Khine et al., 2021; Sun et al., 2023). This finding suggests that extrinsic variables, such as living routines, exert a far greater influence on *Giardia* infection than the sex of the host (Šmit et al., 2023). Similarly, the age-independent pattern observed in our study aligns with previous research (Khine et al., 2021; Li et al., 2019; Nahar et al., 2026). While several studies have reported a higher prevalence of *Giardia* in young animals under 12 months of age due to an immature immune system and a lack of acquired immunity (Kurnosova et al., 2024; Nguyen et al., 2018; Sun et al., 2023), comparable prevalence rates are also reported for older dogs, likely due to reinfection or persistent subclinical infections (Hamnes et al., 2007). In the surveyed rural communes, improper hygiene and unrestricted conspecific contact create an ongoing infection risk for adult dogs. Although puppies possess immunological vulnerabilities, they typically roam less frequently than adults, thereby reducing their relative risk for direct contact with other dogs, potentially explaining the lack of an age association. These results support the conclusion that management practices dictate *Giardia* infection dynamics far more than individual host characteristics (Nahar et al., 2026).

The present study was unable to record specific treatment timelines and anthelmintic products due to inherent recall variability. In addition, the single cross-sectional fecal sampling conducted during the dry season may underestimate the true prevalence, given the intermittent shedding patterns of *Giardia* cysts and *Cryptosporidium* oocysts. Assemblage determination for *G. duodenalis* relied solely on the highly conserved SSU rRNA locus. Although multilocus genotyping was attempted, amplification was unsuccessful. This is a widely recognized diagnostic challenge, as the low cyst shedding typical of asymptomatic dogs frequently yields insufficient DNA to amplify single-copy targets (Gil et al., 2017; Sommer et al., 2018). Future studies would benefit from longitudinal sampling designs and multilocus genotyping targeting single-copy genes, such as triosephosphate isomerase (*tpi*), glutamate dehydrogenase (*gdh*), and beta-giardin (*bg*), to establish more precise prevalence rates, enhance genotyping resolution, elucidate transmission dynamics, and evaluate the effect of seasonality on protozoan transmission. As molecular epidemiological data for companion animals in Vietnam remain sparse, conducting surveillance across other geographic regions is highly recommended to build a comprehensive understanding of these parasites nationwide.

This study provides updated molecular epidemiological and ecological data on enteric protozoans in owned dogs in Central Vietnam. Molecular detection revealed an 8.0% prevalence of *G. duodenalis* and no detectable *Cryptosporidium* spp. infections. Sequence analysis exclusively identified the canine-specific assemblage D, indicating that these companion animals currently present a negligible zoonotic threat to their owners. The findings suggest a highly host-adapted transmission cycle of canine giardiasis within this rural ecosystem, where widespread environmental contamination appears limited. Rather than being driven by individual host demographics, *Giardia* infection dynamics here are dictated by a critical intersection of biological and management variables: specifically, unrestrained conspecific social interactions among dogs and prolonged cyst viability due to moisture retention following improper bathing practices. Therefore, targeted interventions focusing on implementing proper drying protocols and restricting uncontrolled dog-to-dog interactions are crucial to disrupt this parasite transmission cycle. Integrating these targeted management practices with expanded molecular surveillance will be essential for comprehensively mapping and mitigating protozoan transmission across similar agricultural communities.

## Data Availability

The original contributions presented in the study are included in the article/supplementary material. Further inquiries can be directed to the corresponding author.
